# Water’s Unusual Thermodynamics in the Realm of Physical Chemistry

**DOI:** 10.1021/acs.jpcb.2c05274

**Published:** 2022-08-24

**Authors:** Claudio A. Cerdeiriña

**Affiliations:** Departamento de Física Aplicada, Universidad de Vigo—Campus del Agua, Ourense 32004, Spain

## Abstract

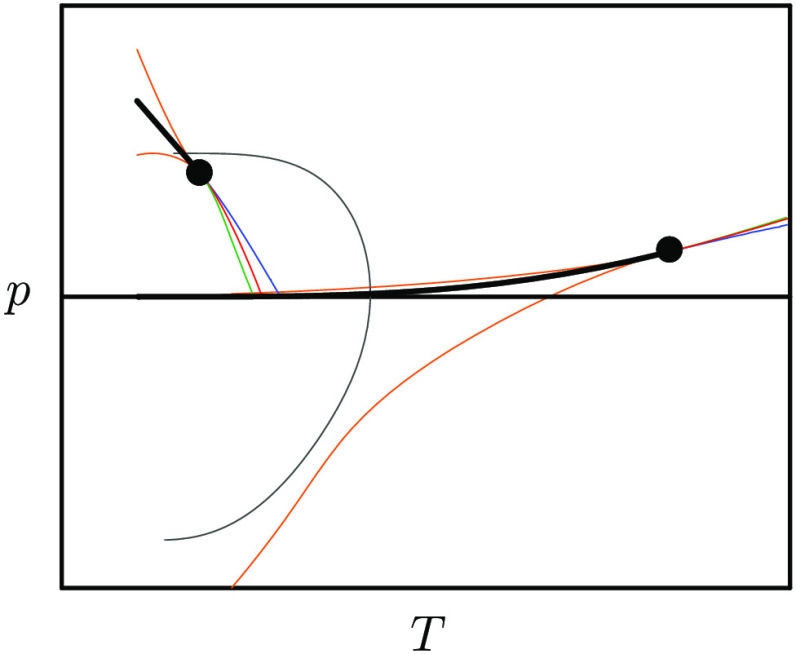

While it is known since the early work by Edsall, Frank and Evans, Kauzmann, and others that the thermodynamics of solvation of nonpolar solutes in water is unusual and has implications for the thermodynamics of protein folding, only recently have its connections with the unusual temperature dependence of the density of solvent water been illuminated. Such density behavior is, in turn, one of the manifestations of a nonstandard thermodynamic pattern contemplating a second, liquid–liquid critical point at conditions of temperature and pressure at which water exists as a deeply supercooled liquid. Recent experimental and computational work unambiguously points toward the existence of such a critical point, thereby providing concrete answers to the questions posed by the 1976 pioneering experiments by Speedy and Angell and the associated “liquid–liquid transition hypothesis” posited in 1992 by Stanley and co-workers. Challenges of this phenomenology to the branch of Statistical Mechanics remain.

## 1 Introduction

Water is an appropriate solvent for a number of chemical reactions and is also the medium in which biological macromolecules exert their functions in vivo. As such, it has been traditionally important to Chemistry and Biology, while it is also so for the Climate and Earth Sciences since water covers almost three-quarters of the planet’s surface. During the past decades, it has acquired increasing relevance to Physics. While the quantum-mechanical description of the water molecule is fairly accurate since long ago, it is the unusual thermodynamic behavior of the bulk liquid that is become a topic of intense research.

**Figure 1:**
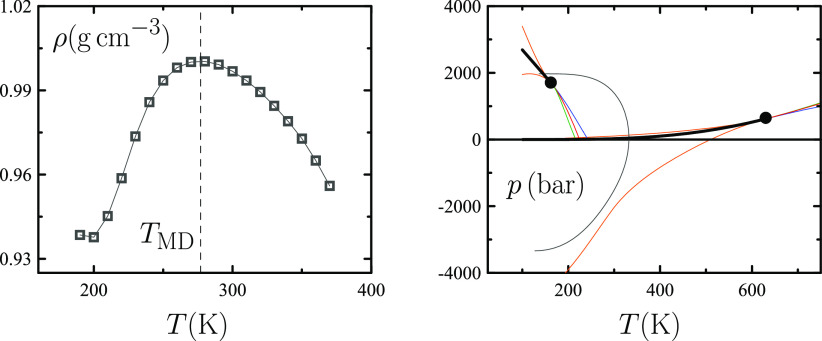
(Left) Density ρ of the TIP4P/2005 force field of water exhibiting a maximum at the temperature *T* = *T*_MD_ along an isobar of pressure *p* = 1 bar.^1^ Note that TIP4P/2005 is known to reproduce water’s experimental ρ(*T*) curve accurately. (Right) Phase diagram of fluid water in the *p*–*T* plane as obtained from the model of ref (1). The two thick black lines are binodal curves representing two-phase coexistence, the orange lines being the associated spinodals. Each binodal terminates at a critical point, gas–liquid at high temperatures, liquid–liquid at low temperatures. Lines of temperatures of maxima along isobars for the isothermal compressibility κ_*T*_ (thin, blue), the isobaric specific heat *c*_*p*_ (thin, green), and the opposite of the isobaric thermal expansivity α_*p*_ (thin, red) emanate from each critical point. Also drawn is the “*T*_MD_ line” (thin, gray) characterizing the temperatures of maximum density at distinct pressures.

This unusual thermodynamics entails the maximum of the density ρ of the stable liquid phase as a function of temperature *T* along isobars of moderate pressure *p*, occurring at *T*_MD_ ≈ 277 K for *p* = 1 bar as Figure 1 illustrates on the basis of information included in ref (1). It also comprises the sharp rises in the magnitude of the isothermal compressibility κ_*T*_, the isobaric specific heat *c*_*p*_, or the isobaric thermal expansivity α_*p*_ as *T* is lowered below the freezing point while water is maintained as a metastable, supercooled liquid. Such an enhanced thermodynamic response of supercooled water led 30 years ago to the hypothesis that it displays a second, liquid–liquid critical point, with the ultimate implication that a one-component fluid can exist as a liquid in more than one form: see Figure 1 for a schematic representation of the corresponding phase diagram in the *p*–*T* plane.

In this context, a first objective here shall be to show what is the latest evidence highlighting that the unusual temperature dependence of water’s density underlies the likewise unusual thermodynamics of aqueous solvation of nonpolar solutes and even certain aspects of the thermodynamics of protein folding. The second part of this Perspective is devoted to the significant support the existence of water’s second critical point has gained over the past few years and to the questions it still poses on the ground of Statistical Mechanics. A few remarks on other lines of research related to the peculiar physical behavior of liquid and supercooled water are finally made.

## 2 Water As a Solvent

### Aqueous Solvation

It has long be recognized that the excluded-volume effects associated with molecular cores are dominant as the solvation (or insertion) of a solute molecule in a solvent-rich phase is concerned. This is the reason standard theories of solvation of small hard spheres, such as Scaled Particle Theory, work reasonably for solvation in water. Such theories prescribe that the scaled solvation free energy , with *k*_*B*_ the Boltzmann constant, varies with *T* and *p* much as the solvent’s ρ does. Then, associated with water’s isobaric ρ(*T*) maximum is a  maximum that implies a minimum of the solubility of solute in solvent as measured by the Ostwald absorption coefficient .

**Figure 2:**
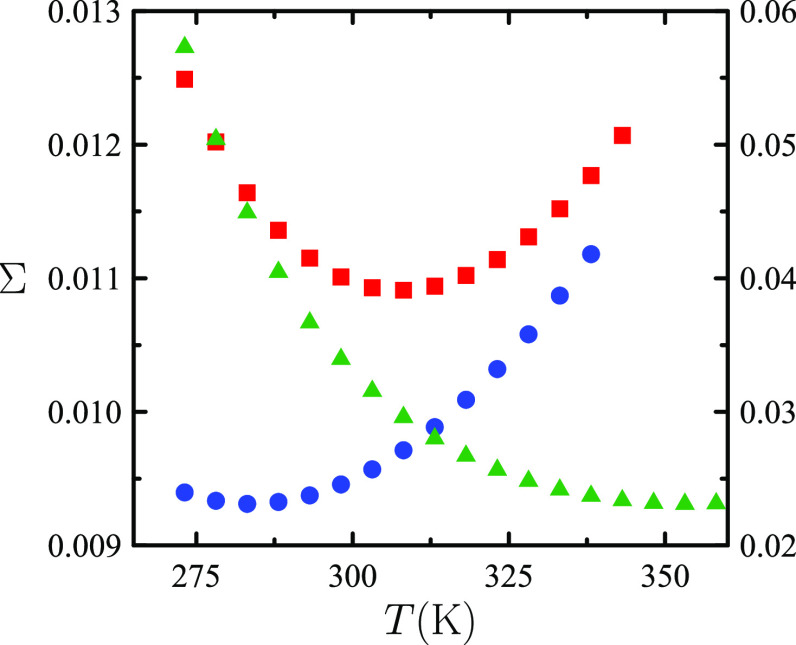
Reprinted with permission from ref (3). Copyright 2018 American Chemical Society. Experimental data of the solubility of solute in solvent as measured by the Ostwald absorption coefficient Σ as a function of temperature *T* for helium (blue), neon (red), and methane (green) in water. The left axis sets the scale for helium and neon while the right one does it for methane. As explained in refs (2 and 3), the Σ(*T*) minimum occurs at *T* = *T*_MD_ when the isochoric solvation energy *u*_V_^*^ vanishes. Accordingly, the temperature of the Σ(*T*) minimum of helium is the closest to *T*_MD_ ≈ 277 K since that solute is the one with the smallest |*u*_V_^*^| value.

Extensive simulation data confirm such a theoretical expectation.^2^ Beyond that, solute–solvent attractive interactions as weak as the ones between hydrocarbons or noble gases and water are not expected to change the picture substantially. Accordingly, experimental Σ(*T*) curves in Figure 2 exhibit a minimum that, as the caption explains, originates from water’s ρ(*T*) maximum.^3^ The “solubility minimum”, which has been traditionally regarded as a fingerprint of the unusual thermodynamics of aqueous solvation of nonpolar solutes, appears as a reflection of water’s density maximum.

**Figure 3:**
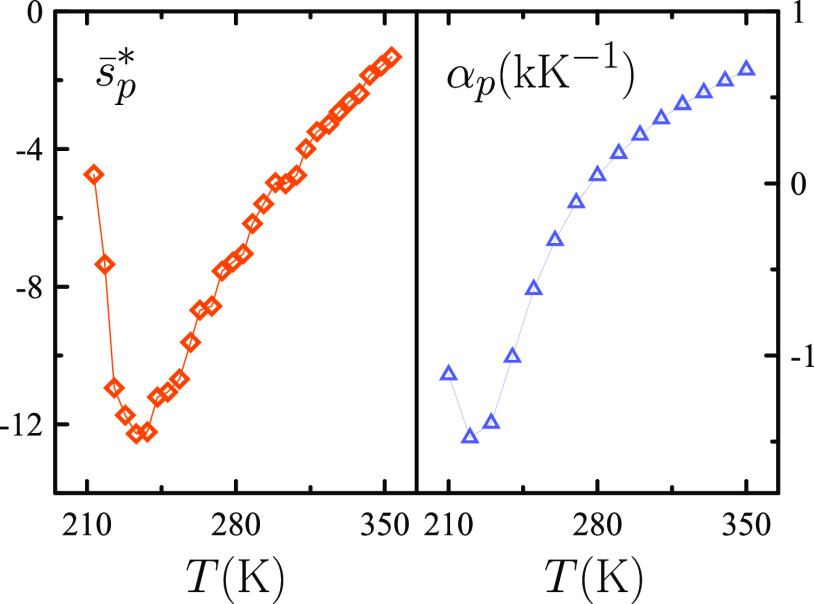
(Left) Dimensionless isobaric solvation entropy , with *k*_*B*_ the Boltzmann constant, as a function of temperature *T* at atmospheric pressure for an “argon-like” solute in TIP4P/2005 water.^4^ (Right) Isobaric thermal expansivity α_*p*_ of TIP4P/2005 water in the same *T* interval.^1^

It is then by no means surprising that a first-order isobaric temperature derivative of  such as the isobaric solvation entropy *s*_p_^*^ reflects the behavior of solvent’s α_*p*_, as the first-order isobaric temperature derivative of ρ(*T*, *p*). Figure 3 illustrates that this is indeed the case.

The *s*_p_^*^(*T*) minimum in Figure 3 implies that the isobaric solvation heat capacity  becomes negative at sufficiently low temperatures. Note that, being a second-order isobaric temperature derivative of , *C*_p_^*^ reflects the curvature of solvent’s ρ(*T*) function. Hence, the change from *C*_p_^*^ > 0 to *C*_p_^*^ < 0 as *T* is lowered is a consequence of the low-temperature convex-to-concave inflection point of TIP4P/2005 ρ(*T*) curve in Figure 1. By the same token, the unusually large and positive *C*_p_^*^ values at near-room temperature, noted in 1935 by J. T. Edsall and historically regarded the first manifestation of the unusual thermodynamics of aqueous solvation of nonpolar solutes, are a natural consequence of water’s relatively large ρ(*T*) curvature around *T* = *T*_MD_.

**Figure 4:**
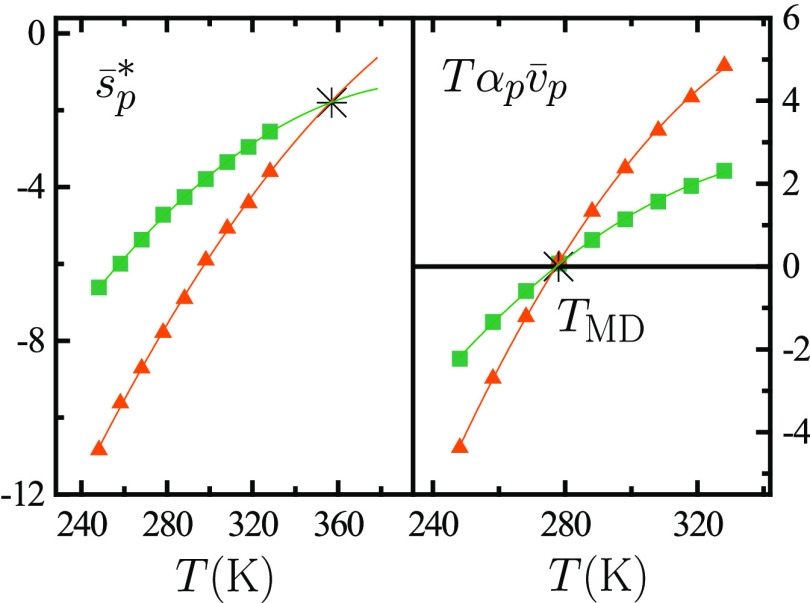
Dimensionless isobaric solvation entropy , with *k*_*B*_ the Boltzmann constant, as a function of temperature *T* at atmospheric pressure for hard-sphere solutes with diameters of 2 Å (squares, green) and 3 Å (triangles, orange) in TIP4P/2005 water.^2^ The right panel shows the values of , where α_*p*_ stands for the solvent’s isobaric thermal expansivity while  with *v*_*p*_ the partial molecular volume and κ_*T*_ the solvent’s isothermal compressibility. The two  curves cross at the temperature of maximum density *T*_MD_ since  is larger the larger the solute while α_*p*_(*T*_MD_) = 0. Such crossing underlies the one of  curves as seen from the exact thermodynamic relation , with  the dimensionless isochoric solvation entropy:  is almost insensitive to *T* changes and increases in magnitude the larger the solute, implying that the crossing of  curves occurs at *T* > *T*_MD_.^2,3^

Water’s density maximum also crucially underlies the crossing of the *s*_p_^*^(*T*) curves of a variety of small solutes of distinct molecular size, a picture often referred to as “entropy convergence.” As Figure 4 explains, the crossing of *s*_p_^*^(*T*) curves originates from the crossing at *T* = *T*_MD_ of the curves corresponding to a contribution to *s*_p_^*^ governing its temperature dependence.

When it comes to solutes with typical dimensions of a few nanometers, water’s unusual thermodynamics manifests in the pattern of solvation in a sharply distinct way.^5^ For such large solutes, Classical Thermodynamics dictates that μ* is made up of a term varying with *T* much like solvent’s liquid–vapor surface tension σ_lv_ and a second one proportional to *p*. Since water’s σ_lv_ is only unusual inasmuch as its value is large relative to that of common liquids, there is no any significant distinction between water and other solvents as the μ*(*T*) behavior along isobars is concerned. On the other hand, along an isochoric path, μ*(*T*) may reflect the isochoric *p*(*T*) minimum associated with the isobaric ρ(*T*) maximum.^6^

### Protein-Folding Thermodynamics

It is known since the work by P. L. Privalov, R. L. Baldwin, and others in the 1970s and 1980s that the isobaric entropy of denaturation of globular proteins displays a convergence picture similar to that observed for the *s*_p_^*^ of small nonpolar solutes in water. This lends support, in accord with W. Kauzmann’s 1959 influential suggestions, to the speculation that the exposure of the nonpolar side chains of amino acids to water upon unfolding effectively parallels the solvation of small nonpolar solutes. It then follows, as just explained in connection with Figure 4, that water’s ρ(*T*) maximum may be a factor for the thermodynamics of protein folding.

**Figure 5:**
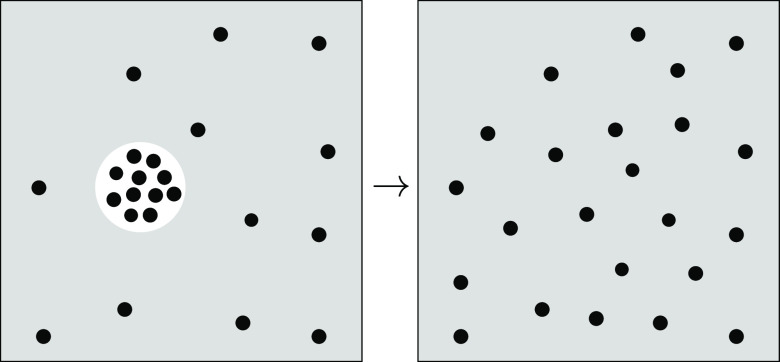
Destabilization of a large cluster composed of small solute molecules (black) in a cavity (white) located at a fixed point in liquid water (gray background). The small solute molecules are interspersed in the aqueous phase upon destabilization, while the whole process is conceived to occur under isothermal–isobaric conditions.

To gauge the plausibility of such a connection, let us assume that underlying the unfolding of a globular protein is the idealized isothermal–isobaric process illustrated in Figure 5.^7^ Thus, we consider the destabilization of a cluster composed by small molecules accommodated in a cavity located at a fixed point in solvent water: the cluster’s individual small molecules are successively transferred from the cavity to the aqueous phase and, after that, the cavity is removed. The first process involves a “small-length-scale” μ* varying with *T* and *p* like *Tρ*, while the second one involves a “large-length-scale” μ* varying with *T* like σ_lv_ and doing it linearly with *p*. Thus, water’s ρ(*T*, *p*) enters in the corresponding overall Gibbs free energy change driving the cluster’s thermodynamic stability according to the Second Law, so that changes in ρ upon *T* and *p* changes may eventually result in changes in the sign of such Gibbs free energy change. Water’s ρ(*T*, *p*) may then be important to denaturation to the extent this simplified model captures the essential aspects of the process.

**Figure 6:**
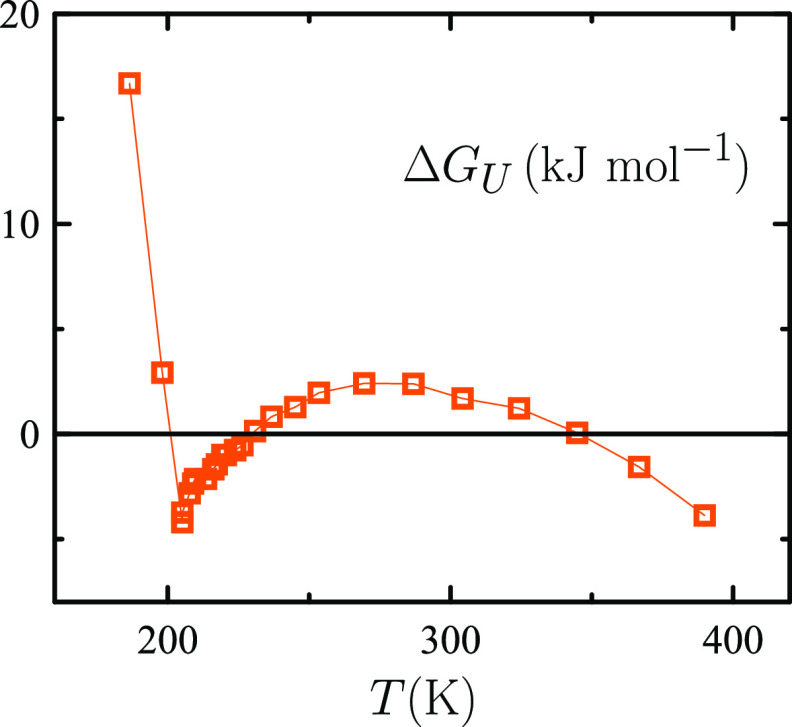
Gibbs free energy of unfolding Δ*G*_*U*_ of Trp-cage miniprotein in TIP4P/2005 water as a function of temperature *T* at atmospheric pressure.^10^

While the relevance of water’s nonstandard ρ(*T*) behavior to protein folding is being increasingly invoked,^8,9^ explicit responses at a quantitative level have begun to emerge. Thus, Figure 6 shows that the temperature dependence of the Gibbs free energy of unfolding Δ*G*_*U*_ for the 20-amino acid model protein Trp-cage in TIP4P/2005 water^10^ correlates with TIP4P/2005 ρ(*T*) behavior in Figure 1. More specifically, starting from an intermediate temperature at which the protein is folded since Δ*G*_*U*_ > 0, “cold denaturation” occurs as temperature is lowered while further cooling leads the protein to refold. That happens just below 200 K, the temperature at which TIP4P/2005 ρ(*T*) exhibits a minimum (see Figure 1).

It is important to note that the observation of the low-temperature refolding of Trp-cage required the use of an enhanced version of the “replica-exchange” technique of Molecular Dynamics.^11^ It is then natural to expect that improved methods leading to augmented computing feasibility and capabilities may help to delve into the relevance of water’s ρ(*T*, *p*) behavior to the protein-folding problem and, by extension, into the question of water’s centrality to Life.

## 3 Water As a One-Component Fluid

### Second Critical Point

Progress in simulation techniques over the last years has allowed to unambiguously prove a liquid–liquid phase transition for ST2 water.^12^ This paved the way for corresponding analyses for TIP4P/2005 and TIP4P/Ice models, which were both found to exhibit liquid–liquid criticality with coordinates *T*_*c*_ ≃ 177 K and *p*_*c*_ ≃ 1750 bar for TIP4P/2005 and *T*_*c*_ ≃ 190 K and *p*_*c*_ ≃ 1725 bar for TIP4P/Ice.^13^ The number of water models with liquid–liquid criticality is actually increasing,^14,15^ while the widely recognized ability of the above two TIP4P variants for reproducing the experimental behavior renders plausibility to the real existence of a second critical point for water.

Consistently, experimental evidence supporting the coexistence of two liquid phases for bulk supercooled water was reported in 2020,^16^ thereby validating the liquid–liquid transition hypothesis put forth in 1992 by H. E. Stanley and co-workers in light of simulations for ST2 water. This achievement involved state-of-the-art experimental techniques allowing to probe bulk water over time scales shorter than the 3-to-50-*μs* characteristic range for ice crystallization. The conditions at which coexistence was observed were quoted to range from 195 to 215 K and from ambient pressure up to 3500 bar. Further progress entails determining the critical coordinates of water’s second critical point accurately.

Elucidating the nature of the associated “one-component liquid–liquid critical behavior” is another line of inquiry. It is generally assumed on theoretical grounds that water’s second critical point belongs to the universality class of the three-dimensional Ising model. A confirmation of such an expectation comes from the detailed simulation analysis of ref (13), which yielded ν ≈ 0.63 and γ ≈ 1.26 for critical exponents comparing favorably with the Ising-3D accepted values ν ≈ 0.63 and γ ≈ 1.24. Corresponding experimental work on critical behavior is naturally demanded.

The wealth of evidence from theory and simulations indicates that the liquid phase of lower density is more ordered and thus has lower entropy, implying from Clapeyron equation that the coexistence line has a negative slope in the *p*–*T* plane. As Figure 1 illustrates, such a negatively sloped coexistence line is continued toward higher temperatures and lower pressures in the one-phase region, thereby defining a so-called “Widom line” that bifurcates into lines of extrema of κ_*T*_, *c*_*p*_, and α_*p*_. Certainly, κ_*T*_(*T*), *c*_*p*_(*T*), and −α_*p*_(*T*) maxima along *p* < *p*_*c*_ isobars are closely associated with the true divergences of these properties at criticality. But experimental evidence confirming the existence of such maxima was not reported until recently.

Explicitly, κ_*T*_(*T*) has been found to display a maximum at atmospheric pressure around 230 K^17^ and *c*_*p*_(*T*) around 229 K.^18^ These findings may again be considered as quite meritorious inasmuch as they entail performing measurements at temperatures lower than the 232 K ice homogeneous nucleation temperature, at which rapid crystallization prevents exploring the supercooled liquid phase in typical experiments. Doubtless, this represents real advance following up on the groundbreaking experimental κ_*T*_(*T*) data for supercooled water reported in 1976 by R. J. Speedy and C. A. Angell.

**Figure 7:**
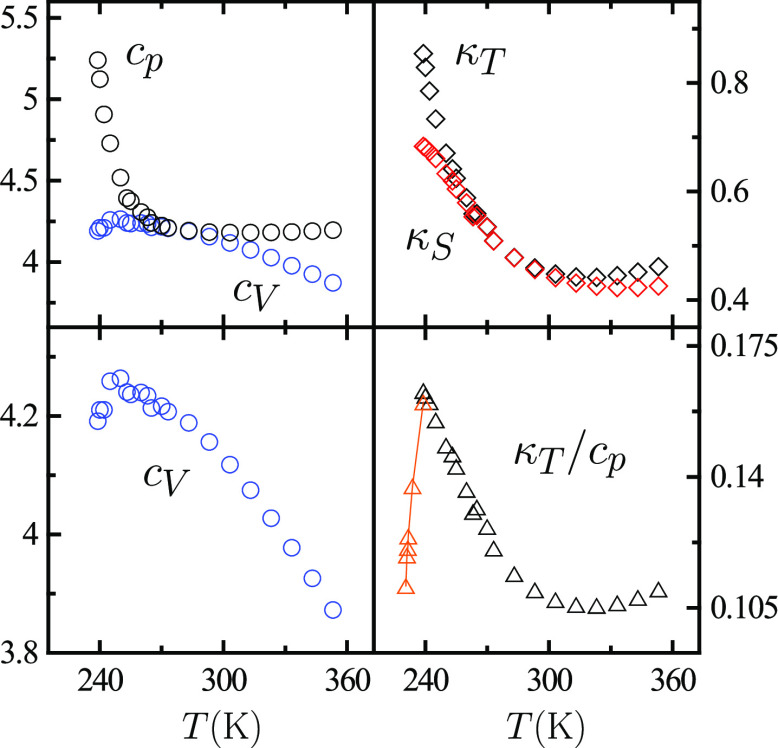
Experimental data for the specific heats, isochoric *c*_*V*_ (circles, blue) and isobaric *c*_*p*_ (circles, black), the compressibilities, isentropic κ_*S*_ (diamonds, red) and isothermal (diamonds, black), and the κ_*T*_/*c*_*p*_ ratio (triangles) of liquid and supercooled water as a function of temperature *T* at atmospheric pressure. Specific heats are in J K^–1^ g^–1^ and compressibilities in GPa^–1^. All properties are represented from 239 to 353 K as obtained from classical sources (Speedy and Angell, etc.). The latest κ_*T*_ and *c*_*p*_ data^17,18^ have been employed to extend the range of κ_*T*_/*c*_*p*_ values plotted down to 230 K (orange), with the temperature correction of ref (18) being adopted for κ_*T*_ data of ref (17).

Besides maxima for the “strongly diverging” κ_*T*_ and *c*_*p*_, maxima for the “weakly diverging” isochoric specific heat *c*_*V*_ and isentropic compressibility κ_*S*_ might exist. Figure 7 shows that the *c*_*V*_(*T*) curve exhibits a shallow maximum at ca. 250 K, while κ_*T*_/*c*_*p*_ displays a relatively sharp one around 239 K. This implies in light of the exact thermodynamic relation κ_*S*_ = *c*_*v*_κ_*T*_/*c*_*p*_ that κ_*S*_(*T*) may mostly reflect κ_*T*_(*T*)/*c*_*p*_(*T*) behavior, so that a κ_*S*_(*T*) maximum near 239 K, that is, above the ice homogeneous nucleation temperature, is to be expected. Experimental κ_*S*_(*T*) data are consistent with such expectation, while the increase of the temperatures of maxima in the sequence *c*_*p*_–κ_*T*_–κ_*S*_–*c*_*V*_ is in accord with thermodynamic constraints associated with a second critical point.^19^

### Statistical Mechanics

While the virtually impenetrable cores of molecules make all liquids to have a similar microscopic structure at sufficiently high pressures, water’s molecular distribution differentiates strikingly from that of other liquids as the pressure is lowered down to moderate (and even negative) values. Thus, at the triple point, the packing fraction *ρv*_vdW_—with *v*_vdW_ standing for the so-called van der Waals volume—is ∼38% for water and ∼60% for neon, argon, xenon, or krypton. The latter four are known to pertain to a broad class of liquids often referred to as “simple liquids.” Liquid water can be losely packed relative to simple liquids and, as such, is considered a representative member of the class of “empty liquids,” as patchy colloids are too.^20^

Simple liquids are understood from the classical interpretation of van der Waals theory due to H. C. Longhet-Higgins and B. Widom, which stresses that the packing effects inherent to the hard part of the pair potential largely determine the liquid’s structure while attractive forces enter as a perturbation. This picture is, however, incapable to sustain a *ρv*_vdW_ value as low as the one of liquid water. It is widely recognized that van der Waals theory breaks down for hydrogen-bonded liquids such as water or alcohols, and while standard theories for molecular association indeed work for alcohols and can even lead to arbitrarily low *ρv*_vdW_ values for the liquid phase, they are still unable to reproduce the unusual thermodynamics of water.^20^

Certainly, the existing theories of association do not capture the special features of hydrogen bonding in water, which is known to generate “ice-like” local structures with a high volume per particle that is incompatible with close packing. A key underlying feature is the existence of an “optimal network forming density” preserving every ice-like structure.^20^ This is consistent with phenomenological approaches identifying the fraction of ice-like structures as the order parameter of the liquid–liquid transition.^21^

Such water’s genuine microscopic features can be implemented in a spin-1, three-state model^1^ pertaining to the Blume–Emery–Griffiths class of Ising-like models formulated long ago and exploiting the concept of “local” volume fluctuations introduced recently by M. E. Fisher in another context. The model characterizes the local energetic, entropic, and volumetric effects associated with ice-like order, whose spatial propagation is then characterized by the “Ising machinery”. While placing full fluid-water phenomenology in the Ising paradigm, this three-state model reduces in the liquid–liquid critical region to a spin-^1^/_2_, two-state version^22^ whose exact solubility allows exploring the nature of liquid–liquid criticality.

Despite progress, a statistical-mechanical theory of liquid water as satisfactory as the one of simple liquids relying on the ideas of van der Waals is still lacking. Further advance on this problem is yet to come.

## 4 Concluding Remarks

Beyond nonpolar solvation, the peculiarities of liquid water may be relevant to a number of classical problems in the area of aqueous solutions that are still a matter of considerable attention. Thus, the evolution of the density maximum upon the addition of solutes has acquired a renewed interest.^23,24^ This is also the case for the structural effects around solutes, as envisioned by H. S. Frank and M. W. Evans in 1945 and recently correlated with water’s ice-like order with the aid of advanced spectroscopic techniques.^25^ Furthermore, careful simulations yielding reliable values for the osmotic second virial coefficient and exact thermodynamic relations involving such property^26^ suggest that water’s unusual thermodynamics may affect the forces between nonpolar solute molecules mediated by water, which are predominantly attractive at high temperatures and repulsive at supercooling conditions. One may also wonder to which extent is the joint description of size and ion-specific effects in aqueous solutions of electrolytes^27,28^ related to water’s unusual dielectric constant entering in the electrostatic part of the solvation free energy as described by the Born model. This is just to mention but a few examples of topics that may eventually bring some attention in connection with water’s thermodynamics.

Research on water’s unusual physical behavior itself is actually expanding in a number of directions. Experimental observation of the one-component liquid–liquid phenomenology for other substances than water is being reported from experiment^29^ and simulation.^30^ Water is not only unusual from the point of view of thermodynamics, yet structural and transport properties also exhibit a peculiar behavior that is the subject of current intense investigation.^31,32^ Likewise, the existence of two glassy states for water has being stimulating vigorous work over the past 35 years in order to determine whether underlying such amorphous states are ice polymorphs or water’s two liquid forms.^33^ Extension of these issues to supercooled aqueous solutions of a variety of ionic and nonionic solutes has opened a wide window to experiment,^34^ which together with molecular simulation is called upon to trigger further progress. In general, the amount of research on these and related topics is growing quickly, and the remaining open questions and presumably upcoming extra challenges shall defy additional efforts.
